# A comparison of the portfolio low-carbohydrate diet and the ketogenic diet in overweight and obese women with polycystic ovary syndrome: study protocol for a randomized controlled trial

**DOI:** 10.1186/s13063-023-07569-6

**Published:** 2023-08-09

**Authors:** Maryam Sharifi Najafabadi, Jalal Moludi, Yahya Salimi, Amir Saber

**Affiliations:** 1 Student Research Committee, School of Nutritional Sciences and Food Technology, Kermanshah University of Medical Sciences, Kermanshah, Iran; 2 Department of Nutritional Sciences, School of Nutritional Sciences and Food Technology, Across From Farabi Hospital, Kermanshah University of Medical Sciences, Isar Sq., P.O. Box 6719851552, Kermanshah, Iran; 3 Social Development & Health Promotion Research Center, Health Institute, Kermanshah University of Medical Sciences, Kermanshah, Iran

**Keywords:** Polycystic ovary syndrome, Portfolio low-carbohydrate diet, Ketogenic diet

## Abstract

**Background:**

Polycystic ovary syndrome (PCOS) is one of the most frequent endocrine disorders among women of fertile age. Women with PCOS manifest clinical symptoms like menstrual dysfunction, hirsutism, insulin resistance, and hyperinsulinemia. As excessive amounts of insulin levels directly increase ovarian production of androgens, hyperinsulinemia and insulin resistance are considered as the pathogenesis factors of PCOS. The portfolio low-carbohydrate diet (PLCD) is a plant-based diet with 40% carbohydrates combined with five cholesterol-lowering foods and nutrients. On the other hand, the ketogenic diet (KD) is a nutritional protocol with 10% carbohydrates. The purpose of this study is to determine whether PLCD or KD is more effective in alleviating PCOS symptoms.

**Methods:**

Forty-six overweight or obese women diagnosed with PCOS will be randomly stratified to receive either PLCD or KD for 8 weeks. Measures related to anthropometric and body composition, glucose, and insulin level, HOMA-IR, sex hormones, lipid profile, quality of life, dietary intake, physical activity, and Ferriman-Gallwey score of all participants will be accessed before and after the intervention.

**Discussion:**

Since the first line treatment of PCOS is lifestyle adjustment including diet control and exercise, there has not been determined the optimal diet for this population of women yet. Hence, the goal of conducting this study is to determine whether the PLCD or the KD could have more advantageous effects on attenuating PCOS manifestations. The result of this investigation will give us new insight into curing this disease and will provide evidence-based recommendations for prescribing an optimal diet for PCOS women.

**Trial registration:**

IRCT20200912048693N3, Trial registered 2022–12-14.

https://www.irct.ir/trial/67548

## Introduction

### Background and rationale {6a}

Polycystic ovary syndrome (PCOS) is the most common endocrine–metabolic disorder, with a prevalence of 4–20% depending on the diagnostic criteria used, among reproductive-aged women [1]. The clinical manifestations of PCOS include menstrual disturbances like oligomenorrhea, amenorrhea, prolonged irregular menstrual bleeding, and signs of androgen excess such as hirsutism and acne as well as alopecia [2]. A diagnosis of PCOS is when at least two of three Rotterdam criteria are presented: (i) clinical hyperandrogenism (such as hirsutism, acne, seborrhea, and alopecia) and/or high circulating androgens levels; (ii) presence of ovarian cysts assessed by ultrasound examination; and (iii) oligo-amenorrhea with oligo-anovulation [3]. Based on these three key parameters of PCOS, four phenotype classifications have been identified [4] (Table 1). As it is illustrated, phenotype A is the most severe, while phenotype D is the least one along an axis of metabolic and ovarian dysfunction. According to a study, phenotypes A, B, and C are endocrine-metabolic syndromes, while phenotype D is non-hyperandrogenic PCOS which represents evidence of both oligo-anovulation and ovarian cysts [5].

**Table 1:** Four phenotype classifications of PCOS according to Rotterdam criteria. As it has shown, phenotype A (classic PCOS) represent all manifestations of PCOS and is the most severe type, whereas phenotype D (non-hyperandrogenism PCOS) manifest neither hyperandrogenism, nor insulin resistance

Features	Phenotype A	Phenotype B	Phenotype C	Phenotype D
Clinical hyperandrogenism	✔	✔	✔	✗
Presence of ovarian cysts	✔	✗	✔	✔
Oligo- or anovulation	✔	✔	✗	✔

✔: Feature present

✘: Feature absent

Despite its etiology is not completely understood, studies indicate that genetic, environmental, metabolic, and endocrine factors all contribute to the pathogenesis of PCOS [6]. Environmental factors are related to both obesity and lifestyle habits [7], while endocrine contributor associated with insulin resistance and hyperinsulinemia [8]. However, approximately 75% of PCOS patients are overweight and/or obese; central obesity is observed in both normal and overweight women [9]. Furthermore, central obesity led to the progression of metabolic syndrome, defined as a tendency of hyperinsulinemia, insulin resistance, and dyslipidemia [10]. Excessive insulin production is directly related to the increased ovarian production of androgens and PCOS manifestations [11]. Women with PCOS also indicate an atherogenic lipid profile, with elevated levels of low-density lipoprotein, cholesterol, and triglycerides, accompany by reduced levels of high-density lipoprotein [12]. Although there has not been a certain treatment for this disease, lifestyle modification, such as diet and exercise, is recommended as the first-line therapy for women with PCOS [13]. Studies demonstrated a 5–10% weight reduction will not only reduce insulin and testosterone levels but also improve ovulatory function and pregnancy rates [14].

The portfolio low-carbohydrate diet is a plant-based diet with 40% carbohydrate, 20% protein, and 40% fat. The Portfolio diet was developed in the early 2000s as a “portfolio” of five cholesterol-lowering foods and nutrients (plant protein, viscous fiber, nuts, and phytosterols) [15]. The Portfolio diet has lower saturated fat and cholesterol with five food components added to the diet, including plant protein from soy products or dietary pulses, viscous soluble fiber from oats, barley, certain fruits, nuts, and plant-based monounsaturated fats (MUFAs) in forms of olive and canola. Studies indicate that the Portfolio diet has positive effects on lipid profile and increase high-density lipoprotein (HDL) cholesterol [16]. On the other hand, studies confirm that following a low carbohydrate diet indicates improvements in waist circumference, fasting glucose and, serum insulin as well as weight in women with polycystic ovary syndrome [17]. In this way, Thomson and colleagues indicated that a low carbohydrate diet with 30% protein, 40% carbohydrate, and 30% fat on 94 overweight and obese women with PCOS during 20 weeks could decrease fat mass and fat-free mass 3 and 2 kg, respectively [18]. The ketogenic diet is a nutrition protocol of high-fat (70–80%), very low-carbohydrate (5–10%) diet which represent beneficial effects in a number of diseases, including obesity, neurological disorders, type 2 diabetes mellitus, cancer, and PCOS [19, 20]. The role of a ketogenic diet on PCOS is through the reduction of the amount of circulating glucose and insulin, which results in both declining glucose oxidation and increasing fat oxidation [21]. While many studies illustrate, KD significantly decreased body weight, glucose, and insulin levels, triglycerides, total cholesterol, LH, testosterone, and DHEA levels with a significant improvement of HOMA-IR. Furthermore, estradiol, progesterone, SHBG, and HDL increased [22, 23].

Despite there not being indicated a certain diet for PCOS therapy [24], studies revealed low-carbohydrate diets are more effective than standard hypocaloric diets, due to their regulatory effects on insulin secretion [17]. For instance, in 2022, a clinical trial was conducted to determine the therapeutic effects of the Mediterranean low-carbohydrate diet (MED/LC) (20% of carbohydrate) with a low-fat diet (30% fat) in 72 overweight PCOS patients for 12 weeks. Their results illustrated that the MED/LC was more effective in modifying anthropometric parameters, reproductive endocrine levels, insulin resistance levels, and lipid levels compared with the low-fat diet and recommended the MED/LC diet for the treatment of overweight patients with PCOS [25]. Another investigation through 14 overweight PCOS women revealed a significant reduction of anthropometric indices, insulin, glucose level, HOMA-IR, and lipid profile, as well as reproductive endocrine hormones after following a ketogenic diet. It also considered the KD as a valuable non pharmacological treatment for PCOS patients [21].

As it has been shown, studies have confirmed the positive effect of both low-carbohydrate and ketogenic diets on PCOS because of their lower amount of carbohydrates and the consequence of lower levels of insulin secretion in comparison with the conventional diet. Consequently, these two diets could effectively reduce hyperandrogenism as well, which could be beneficial for patients with phenotypes A, B, and C of PCOS. However, for phenotype D, which lacks hyperandrogenism, these dietary interventions do not hold therapeutic rationale due to the distinct etiology. Overall, due to our knowledge, there has not been any study to represent which of these two diets is more effective on treating PCOS. Therefore, the aim of the present study is to determine whether the PLCD or the KD could have more beneficial effects for improving PCOS by measuring anthropometric indices, hormonal status, lipid profile, quality of life, dietary intake, physical activity, and Ferriman-Gallwey score.

### Objectives {7}

1. To determine the effect of PLCD compared to the KD on anthropometric (height, weight, waist circumference, hip circumference, waist-to-hip ratio, and body mass index), body composition indices (fat body mass, lean body mass), and basal metabolic rate (BMR) in overweight and obese women with PCOS
2. To determine the effect of PLCD compared to the KD on the sex hormonal (LH, FSH, DHEA) status in overweight and obese women with PCOS
3. To determine the effect of PLCD compared to the KD on hirsutism status in overweight and obese women with PCOS
4. To determine the effect of PLCD compared to the KD on lipid profile in overweight and obese women with PCOS
5. To determine the effect of PLCD compared to the KD on insulin, and glucose level, as well as HOMA-IR in overweight and obese women with PCOS
6. To determine the effect of PLCD compared to the KD on the quality of life in overweight and obese women with PCOS

### Trial design {8}

This is a parallel-arm, open, stratified randomized clinical trial. Forty-six overweight or obese women diagnosed with PCOS will be randomly stratified to receive either PLCD or KD for 8 weeks.

## Methods: participants, interventions, and outcomes

### Study setting {9}

**Fig.1:**
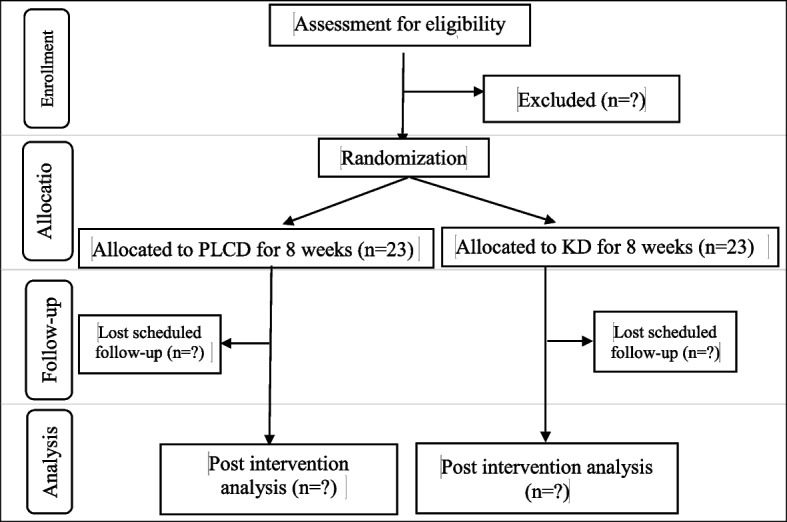
CONSORT flow diagram of study design for portfolio low-carbohydrate and the ketogenic diet in women with polycystic ovary syndrome

This is a parallel-arm, open, stratified randomized clinical trial. The protocol was approved by the Medical University of Kermanshah Research Ethics Board (IR.KUMS.REC.1401.404). Furthermore, the trial was registered on irct.ir (IRCT20200912048693N3) on 2022 December 14. This study will be conducted in Fatahi Clinic of Kermanshah, Iran. As the study design is detailed in Fig. 1, potential patients firstly will be invited to Fatahi Clinic where a registered dietitian (RD) and an obstetrician-gynecologist will visit them to assess whether they have eligibility to enter this trial. Also, participants will provide written informed consent after the study was fully explained. Then, desirable participants will return to the Reference Laboratory of Kermanshah to undergo baseline blood tests including FBS, insulin level, and lipid profile, as well as sex hormones during the beginning of the follicular phase (days 3–8 of the menstrual cycle). After that, women will return to the Fatahi Clinic to have a dietary consult with an expert dietitian and randomly receive one of the diets. They will also be encouraged to return 2 months later to determine dietary adherence and undergo end of intervention testing. During these 2 months, the dietitian will call each patient every 2 weeks to follow-up with them and, if necessary, provide them with appropriate recommendations.

### Eligibility criteria {10}

#### Inclusion criteria

The inclusion criteria are as follows:

1. Written informed consent
2. Diagnosis with PCOS according to the Rotterdam Criteria by an obstetrician-gynecologist
3. Age 18–45 years
4. BMI ≥ 25 kg/m^2^

#### Exclusion criteria

The exclusion criteria are as follows:

1. Currently pregnancy or lactation
2. Taking anti-psychotic or anti-seizure medication due to their induce effect on insulin resistance
3. Following a specific nutritional diet or hypocaloric diet in the last 3 months
4. History of diabetes or other endocrine disorders (thyroid dysfunction and adrenal disorders)
5. Using medications that impact carbohydrate or lipid metabolism (oral contraceptive pills, antiepileptic, antipsychotics, statins, and fish oil)
6. Using fertility-enhancing or weight loss medications (except metformin)
7. History of hormonal therapy and/or insulin-sensitizers within the previous 2 months
8. History of hepatic and renal disorders, hypertension, diagnosed anemia, severe respiratory disease (asthma and chronic bronchitis), and heart disease

### Who will take informed consent? {26a}

The data of all of the participants will be kept in the Kermanshah University, and just investigators of this trial will have access to them.

### Additional consent provisions for collection and use of participant data and biological specimens {26b}

On the consent form, participants will be asked if they agree to use their data should they choose to withdraw from the trial. Participants will also be asked for permission for the research team to share relevant data with people from the universities taking part in the research or from regulatory authorities, where relevant. This trial does not involve collecting biological specimens for storage.

## Interventions

### Explanation for the choice of comparators {6b}

Participants will randomly stratify to the PLCD or the KD designed by a qualified dietician to follow for 8 weeks.

## Intervention description {11a}

### Dietary intervention

A PLCD is a plant-based diet with a macronutrient distribution of 40% carbohydrate, 20% protein, and 40% fat. The Portfolio diet consists of four food components added to the diet (based on a 2000 kcal diet): 50 g plant protein from soy products or dietary pulses such as beans, peas, chickpeas, and lentils; 20 g viscous soluble fiber from oats, barley, psyllium, eggplant, okra, and certain fruit; 45 g nuts (tree nuts or peanuts); and 2 g phytosterols. Later on, a fifth component was joined to the diet, as plant-based monounsaturated fats (MUFAs) in the form of olive, canola, or high oleic sunflower oils (45 g/day) that indicated more improvements of blood lipids, such as high-density lipoprotein (HDL) and cholesterol [26]. So, each diet will be determined according to the prescribed daily calorie and macronutrient intake. On the other hand, the KD contains 10% carbohydrates, 20% protein, and 70% fat. Carbohydrates intake will be < 30 g per day which will be provided by a variety of vegetables as they ate rich in fiber and have low amounts of carbohydrates. Protein intake will be provided through fish, poultry, meat, and egg consumption. Furthermore, lipid components will be consumed in the form of olive oil, sesame, and oilseeds. Unsweetened drinks like herbal teas, tea, and infused coffee will be allowed as they would not induce ketosis. Both groups will take a multivitamin-mineral supplement (Dana Corp. Multi Daily) every day. Each patient will have at least ninety minutes of dietary counseling with a dietitian to educate their diet, be encouraged to have aerobic physical activity, for instance, walking or jogging at least 60 min per day, and be remined to stop continuing the diet if they realized that they are pregnant. To induce patients’ motivation of following their diet, the dietitian will assure them to prescribe a stabilized diet at the end of the study. Furthermore, the dietitian will be readily available to guide and advise individuals via phone, email, and other media.

### Criteria for discontinuing or modifying allocated interventions {11b}

There have not been observed serious complications for PLCD [27]. Furthermore, there have been reported some short-term and long-term complications for the ketogenic diet. Short-term complications are dehydration, hypoglycemia, lethargy, and gestational side effects (diarrhea, nausea, and vomiting), while long-term complications are hypercalcemia, lipid profile change, urolithiasis, gallstone, and hair loss [28]. As the trial studies have been done for a short time ((12 weeks) [21], (6 weeks) [22]) and reported the beneficial effects of the ketogenic diet without any adverse complications, our trial will be conducted for short time (8 weeks) as well. The dietitian will also announce each participant of the complications of their diet and remind them to stop their diet if they feel any adverse effects or discovered they are pregnant.

## Strategies to improve adherence to interventions {11c}

### Dietary monitoring

The dietitian will call each patient every 2 weeks to document their 3-day food records, two working days as well as the weekend, assess and analyze their macronutrient composition and calorie intake to realize how they follow their diet and if necessary, and provide them with appropriate recommendations. In order to ensure that nutritional ketosis will be induced and maintained in women who follow the KD, they will be required to use urinary keto sticks every week and send their picture to the dietitian.

### Relevant concomitant care permitted or prohibited during the trial {11d}

There are no restrictions regarding concomitant care during the trial. Implementing the intervention will not require alteration to usual care pathways (including the use of any medication), and these will continue for both trial arms.

### Provisions for post-trial care {30}

As there has not seen any adverse effects for these two diets, this trial does not have any provisions for post-trial care.

### Outcomes {12}

#### Primary outcomes

The primary outcome of this investigation is to determine the effectiveness of the PLCD compared to the KD in improving PCOS disease symptoms by measuring weight loss before and after the intervention.

#### Secondary outcomes

The secondary outcomes are including body mass index (BMI), fat body mass (FBM), lean body mass (LBM), body fat percentage, waist circumference, insulin level as well as sex hormones (testosterone, LH, FSH, DHEA), lipid profile (cholesterol, triglycerides, low-density lipoprotein (LDL), high-density lipoprotein (HDL)), glucose levels, HOMA index – IR, physical activity, health-related quality of life (SF-36), and Ferriman-Gallwey score, before and after the intervention.

### Participant timeline {13}

**Fig. 2:**
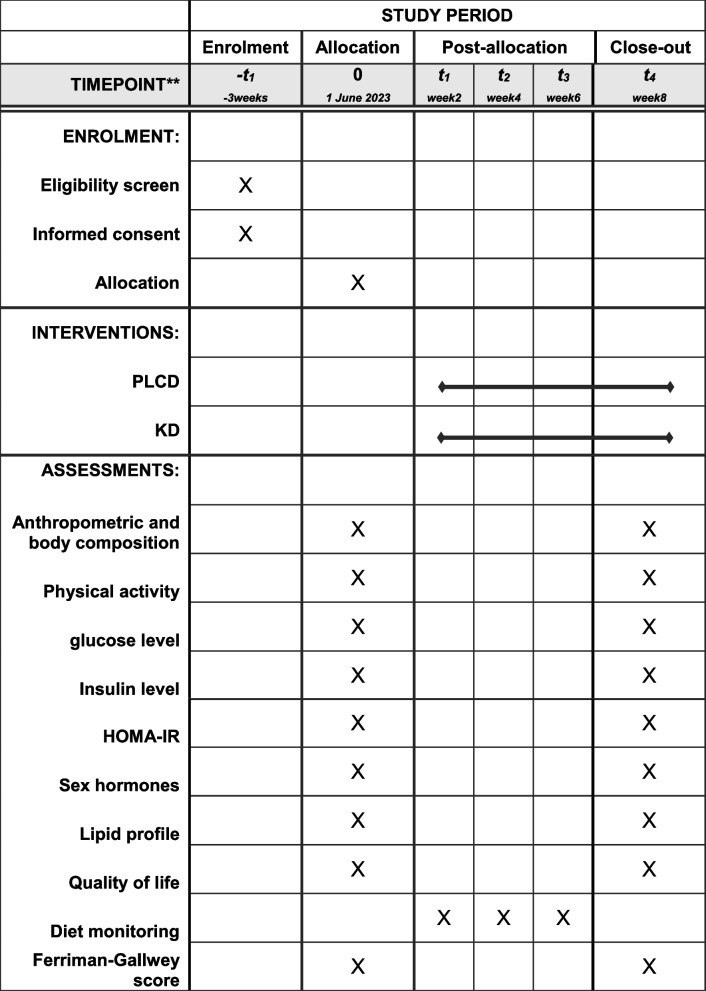
Standard Protocol Items: Recommendations for Interventional Trials (SPIRIT) schedule of enrolment, interventions, and assessments for the duration of the study. PLCD, portfolio low-carbohydrate diet; KD, ketogenic diet; HOMA-IR, homeostatic model assessment-insulin resistance

The participant timeline is shown in Fig. 2.

### Sample size {14}

According to the formula, the standard deviation of weight in the dietary group of PLCD and KD would be equal to μ_1_ = 3.14 (σ_1_ = 2.42) and μ_2_ = 1.66 (σ_2_ = 0.8) respectively. In the following sample with *α* = 0.05, the sample size required to have 80% power to detect a difference of 2 units of change in weights would be 18 people in each group. Considering the possibility of 25% missing people in the follow-up, the sample size increases to 23 people in each group.

$$n=\frac{{\left({z}_{1-\frac{a}{2}}+{z}_{1-\beta }\right)}^{2}+\left({\sigma }_{2}^{1}+{\sigma }_{2}^{2}\right)}{{\left({\mu }_{1}-{\mu }_{2}\right)}^{2}}$$

## Recruitment {15}

### Participant recruitment

We aim to recruit 46 participants (23 individuals in each arm) diagnosed with PCOS using online advertising and posters posted through Motazadi Gynecology and Obstetrics Hospital, healthcare establishments, physician offices, pharmacies, and laser clinics. These people will come from the public population. One of the researchers involved in the study will do the recruitment. Participants will be asked to visit Fatahi Clinics where the screening will be performed. At the first visit, the screening will be carried out by a dietitian and an obstetrician-gynecologist to determine eligible patients according to the inclusion criteria and have a detailed explanation about the study, as well as provide written informed consent.

## Assignment of interventions: allocation

### Sequence generation {16a}

#### Randomization

During the first visit, participants will be randomized to either the PLCD or the KD. Randomization will be conducted by an investigator who is not involved in obtaining or entering participants’ data. The samples will be included in the study with informed consent using a random block method of 6 that will be created by the website https://www.sealedenvelope.com.

#### Concealment mechanism {16b}

The following will be considered in the implementation of the random assignment process: (a) one English letter is assigned to each of the groups: A to the PLCD group, B to the KD group; (b) a sequence for the sample size 23 will be created; (c) for the concealment process of random allocation, 23 non-transparent envelopes and cards (as much as the total sample size) will be prepared. Inside each envelope, 6 cards will be placed in the order of the sequence in each block. Block numbers will be written on each envelope. The name of the desired group will be written on each card in sequence.

#### Implementation {16c}

The investigator who collects the information is unaware of the type of allocation of the samples to the study groups. The participants will be enrolled to the study by both a dietitian and an obstetrician-gynecologist according to the eligible criteria. Then, the dietitian will randomly stratified participants to receive either PLCD or KD.

## Assignment of interventions: blinding

### Who will be blinded {17a}

This trial will not be blinded due to the nature of the intervention.

### Procedure for unblinding if needed {17b}

This trial will not be blinded due to the nature of the intervention.

## Data collection and management

### Plans for assessment and collection of outcomes {18a}

#### Baseline measures

After the screening, the demographic characteristics questionnaire, the physical activity questionnaire (IPAQ), the Ferriman-Gallwey score, and health-related quality of life (SF-36) as well as 3-day food records questionnaires will be completed. Furthermore, all participants will undergo baseline assessments. Biochemical analysis and anthropometric measurements include FBS, insulin level, lipid profile (cholesterol, triglycerides, LDL, and HDL), sex hormones (testosterone, LH, FSH, and DHEA) during the beginning of the follicular phase (days 3–8 of the menstrual cycle), weight, height, weight, waist circumference, hip circumference, waist-to-hip ratio (WHR), and body composition indices (fat body mass (FBM), lean body mass (LBM)). Also, body mass index (BMI) and HOMA-IR will be calculated according to their formula. Serum levels of blood ketone bodies (KBs) will be assessed to ensure they are not in ketosis phase at the beginning of the study.

1. Anthropometric

Body weight, high, waist circumference, and hip circumference will be determined by a proficient research assistant at the Fatahi Clinic. Body weight will be measured with the subject wearing light clothing, shoes, and socks removed, to the nearest 0.1 kg using an electronic scale with bioimpedance analysis (Plus Avis 333 Body Analyzer, China). Hight will be determined to the nearest 1 cm using a well-mounted portable stadiometer (Holtain Ltd, UK), body mass index (BMI) will be calculated in Kg/m^2^, waist circumference will be measured after removing clothes from the abdomen, as the smallest circumference between the lowest rib and the iliac crest on the midaxillary line, the hip circumference will be determined at the level of the widest circumference over the great trochanters, and waist-to-hip ratio will be calculated as waist measures divided by hip measurement [29].

Body composition analysis as muscle mass (MM), body fat mass (FM), percent body fat (PBF), and total body water (TBW) will be expressed in kilograms, while total energy expenditure (TEE) will be reported in Kcal and will be analyzed using an electronic scale with bioimpedance analysis (Plus Avis 333 Body Analyzer, China). All parameters will be measured before and two months after the diet intervention.

2. Blood sampling and collection

After at least 10 h of fasting, 10 ml venous blood sample will be drawn by venous puncture from the bronchial vein of the participants to assess fasting blood sugar (FBS), insulin level, sex hormones, and lipid profile. Luteinizing hormone (LH), follicle-stimulating hormone (FSH), dehydroepiandrosterone sulfate (DHEA), testosterone, and insulin levels will be evaluated by immunochemiluminescent method, blood glucose by the enzymatic method with hexokinase, total cholesterol, high-density lipoprotein (HDL), low-density lipoprotein (LDL), and triglycerides by enzymatic colorimetric assay. The hormonal status evaluation will be performed during the follicular phase of the menstrual cycle, between the first and the seventh day. The Homeostatic Model Assessment (HOMA-IR) index will be calculated according to the formula (insulinemia mU/ml × glycemia mg/dL/450). After the first evaluation, patients will undergo the intervention, until the end of 2 months when they will be re-evaluated in the same way. All biochemical tests will be assessed clinically at the Reference Laboratory of Kermanshah University of Medical Sciences.

3. Ferriman-Gallwey score

Hirsutism is considered as one of the common clinical symptoms of polycystic ovary syndrome which is due to hyperandrogenism. It is estimated that 70% of women with PCOS also manifest hirsutism symptoms. Hirsutism is evaluated by the Ferriman-Gallwey score [30, 31].

4. Quality of life questionnaire

The quality of life questionnaire for women with polycystic ovary syndrome has been validated previously and used to both determine the psychological impact of having PCOS and the effect of participation in the intervention [30, 32]. Thus, it will be used in this study with the same purpose.

## Plans to promote participant retention and complete follow-up {18b}

To induce patients’ motivation of following their diet, the dietitian will assure them to prescribe a stabilized diet at the end of the study. Furthermore, the dietitian will be readily available to guide and advise individuals via phone, email, and other media.

## Data management {19}

### Data management and analysis

The SPSS software (version 19) will be used for data analysis. Initially, the Kolmogorov–Smirnov test will be used to check the normality of the data. Data with normal distribution will be used to compare within-group and inter-group means by Student’s *t*-test. For data that do not have a normal distribution, Wilcoxon and Mann–Whitney tests will be used for intragroup and intergroup comparisons. In addition, the chi-square test will be used to examine qualitative data. The N-IV software will be used to analyze diet data. A *P*-value less than 0.05 will be considered significant.

### Confidentiality {27}

The data of all of the participants will be kept in Kermanshah university and just investigators of this trial will have access to them**.**

### Plans for collection, laboratory evaluation, and storage of biological specimens for genetic or molecular analysis in this trial/future use {33}

At the beginning and end of the intervention, 10 cc of venous blood will take from all patients in the fasting state and in the early follicular phase (days 3–8 of the menstrual cycle), and after centrifugation, the plasma will be stored at – 80 °C until hormonal and biochemical tests. Sampling and specialized tests are carried out in the central laboratory of the Kermanshah University of Medical Sciences under the supervision of physicians and relevant experts.

## Statistical methods

### Statistical methods for primary and secondary outcomes {20a}

Data analysis will be done using SPSS Statistics (version 19, NY, USA). A two-tailed statistical significance will be accepted at *P* < 0.05. Each outcome variable assessed will be analyzed according to diet (PLCD or KD) and time (before and after each diet) using a repeated-measures two-way analysis of variance (ANOVA). Where appropriate, a Student–Newman–Keuls post hoc test will be used to assess the origin of any significant differences detected by the ANOVA models. Variables will be adjusted for potential confounders which will be determined using multiple linear regressions.

### Interim analyses {21b}

No interim analyses will be performed.

### Methods for additional analyses (e.g., subgroup analyses) {20b}

We intend to perform subgroup analysis, such as fat body mass vs. lean body mass, LDL vs. HDL, testosterone vs. luteinizing hormone (LH). We will try to match for most variables across groups. Adjustments will be made for confounding factors like waist circumference, macronutrients consumption, and physical activity.

### Methods in analysis to handle protocol non-adherence and any statistical methods to handle missing data {20c}

An “intention to treat” analysis will be conducted to handle protocol non-adherence. In the case of more than 10% missing for the primary outcome, we plan to impute missing values using the multiple imputation method.

### Plans to give access to the full protocol, participant level-data and statistical code {31c}

This trial does not have any plan for giving access to the full protocol, participant-level data, and statistical code.

## Oversight and monitoring

### Composition of the coordinating center and trial steering committee {5d}

All research team members will collaborate in preparing all the study steps.

### Composition of the data monitoring committee, its role and reporting structure {21a}

All research team members will collaborate in preparing all the study steps.

### Adverse event reporting and harms {22}

As previously mentioned, these two diets do not have serious adverse or harmful effects on short time. It seems that the ketogenic diet demonstrates some adverse events (AEs) such as dehydration, hypoglycemia, lethargy, and gestational side effects (diarrhea, nausea, and vomiting) for a short time and serious adverse effects (SAEs) like hypercalcemia, lipid profile change, urolithiasis, gallstone, and hair loss for long-term intervention. Since our trial will be conducted for a short time (8 weeks), it is predicted to have no SAEs. However, each participant will be informed of the side effects of their diet by a dietitian and will be reminded to stop the diet if they experience any AEs or discover they are pregnant. Moreover, SAEs will be reported to the DMEC, indicating expectedness, seriousness, severity, and causality.

### Frequency and plans for auditing trial conduct {23}

It is anticipated that every week, the project management group will meet to review trial conduct.

Also, the trial steering group as well as the independent data monitoring and ethics committee will meet to review conduct throughout the trial period, once every 2 weeks. In addition, the trial steering group (Iranian Steering Committee for Clinical Trials) as well as the independent data monitoring committee will observe the day-to-day progress of this trial. The ethics committee of the Kermanshah University of Medical Sciences will control the authenticity of the trial. The faculty of Nutritional Sciences and Food Technology is responsible for the scientific requirements.

### Plans for communicating important protocol amendments to relevant parties (e.g., trial participants, ethical committees) {25}

The researchers will conduct the study in compliance with the protocol provided. Any modifications to the protocol including study objectives, study design, eligibility criteria, sample sizes, or significant changes in the study that will impact study conduct, potential benefit, or safety of the study participant will be communicated with the ethical committee.

### Dissemination plans {31a}

In the end, the results of this work will be published as an article.

## Discussion

This trial is designed to determine whether PLCD or KD could have more beneficial effects for improving PCOS disease by measuring anthropometric indices, hormonal status, lipid profile, and quality of life questionnaire. According to the 2018 guidelines of the American Society for Reproductive Medicine (ASRM), the first line of treatment for PCOS is lifestyle adjustment, including diet control and exercise, with the weight control for women suffering from PCOS [33]. According to different studies, dietary interventions have been shown to eliminate clinical symptoms of PCOS, for instance, menstrual disorders, abnormal hormonal indicators, and ovulation [14, 34]. Thus, diet modification could play an important role in improving the clinical symptoms of PCOS. Although there has not been a univocal therapy for PCOS, several studies indicate the importance of macronutrient distribution especially carbohydrates in the diet of women with PCOS due to their effect on insulin secretion from the pancreas [35]. As the KD has the utmost 10% carbohydrate, it activates AMPK and SIRT-1, which have beneficial effects on glucose homeostasis and improving insulin sensitivity [36]. On the other hand, studies have shown that LCD regimens reduce the levels of glucose, IGF-1, IGFBP1, and insulin [17]. In addition, due to the importance of insulin receptors and compensatory hyperinsulinemia in the reduction of androgen excess in PCOS women, LCD could improve hyperandrogenism-related symptoms. Therefore, this novelty study is put forward to specify which diet, PLCD or KD, would have more advantageous effects on PCOS patients. As PCOS women display an atherogenic lipid profile (associated with elevated levels of low-density lipoprotein, triglycerides, and cholesterol, along with reduced levels of high-density lipoprotein), applying the Portfolio diet in combination with a low-carbohydrate diet will provide us with unique data about their effects on lipid profile in comparison with the KD [12]. In addition, this trial is a multi-disciplinary approach that has combined assessments of nutrition, psychology, lipid profile, and reproductive medicine. Hence, the result of the current study could give us a new and comprehensive insight into this multi-faced syndrome and give more information about prescribing therapeutic diets for PCOS patients. The current study will further provide evidence to inform and aid in the future dietary therapeutic management of women with PCOS. As lifestyle adjustment specifically diet has been shown to ameliorate the clinical symptoms of PCOS, there is no consensus on an optimal diet yet [37]. Thus, this trial will provide us with useful information about prescribing an efficient and effective diet with proper control of carbohydrate intake as an essential approach for PCOS women.

## Trial status

This trial was registered on irct.ir (IRCT20200912048693N3) on 2022 December 14. Thus, it is considered to start on 2023 May 20 and will end on 06 January 2023.

## Data Availability

No applicable.

## Abbreviations

BMI: Body mass index
DHEA: Dehydroepiandrosterone sulfate
FBM: Fat body mass
FBS: Fasting blood sugar
FSH: Follicle-stimulating hormone
HDL: High-density lipoprotein
HOMA_IR: Homeostasis model assessment-insulin resistance
KD: Ketogenic diet
LH: Luteinizing hormone
LBM: Lean body mass
LDL: Low-density lipoprotein
MM: Muscle mass
PCOS: Polycystic ovary syndrome
MUFAs: Monounsaturated fats
PLCD: Portfolio low-carbohydrate diet
RD: Registered dietitian
SHBG: Sex hormone-binding globulin
TBW: Total body water
TEE: Total energy expenditure
